# Neuroanatomical Correlates of the Late Positive Potential in Youth with Pediatric Bipolar Disorder

**DOI:** 10.2174/1570159X21666230413104536

**Published:** 2023-05-18

**Authors:** Alessio Simonetti, Marijn Lijffijt, Sherin Kurian, Johanna Saxena, Delfina Janiri, Marianna Mazza, Giulio Carriero, Lorenzo Moccia, Benson Mwangi, Alan C. Swann, Jair C. Soares

**Affiliations:** 1 Menninger Department of Psychiatry and Behavioral Sciences, Baylor College of Medicine, Houston, TX, 77030, USA;; 2 Department of Neuroscience, Section of Psychiatry; Fondazione Policlinico Universitario “Agostino Gemelli” IRCCS, Rome, Italy;; 3 Michael E. DeBakey VA Medical Center, Houston, TX, 77030, USA;; 4 Department of Psychiatry, Texas Children’s Hospital, Houston, TX, 77030, USA;; 5 Department of Neurology and Psychiatry, Sapienza University of Rome, Rome, Italy;; 6 Department of Psychiatry and Behavioral Sciences, University of Texas Health Science Center, Houston, TX, 77030, USA

**Keywords:** Pediatric bipolar disorder, event-related potentials, late positive potential, magnetic resonance imaging, cortical thickness, subcortical volumes

## Abstract

**Background:**

The late positive potential (LPP) could be a marker of emotion dysregulation in youth with pediatric bipolar disorder (PBD). However, the neuroanatomical correlates of the LPP are still not clarified.

**Objective:**

To provide cortical and deep gray matter correlates of the LPP in youth, specifically, youth with PBD.

**Methods:**

Twenty-four 7 to 17 years-old children with PBD and 28 healthy controls (HC) underwent cortical thickness and deep gray matter volumes measurements through magnetic resonance imaging and LPP measurement elicited by passively viewing emotional faces through electroencephalography. T-tests compared group differences in LPP, cortical thickness, and deep gray matter volumes. Linear regressions tested the relationship between LPP amplitude and cortical thickness/deep gray matter volumes.

**Results:**

PBD had a more pronounced LPP amplitude for happy faces and a thinner cortex in prefrontal areas than HC. While considering both groups, a higher LPP amplitude was associated with a thicker cortex across occipital and frontal lobes, and with a smaller right globus pallidus volume. In addition, a higher LPP amplitude for happy faces was associated with smaller left caudate and left globus pallidus volumes across both groups. Finally, the LPP amplitude correlated negatively with right precentral gyrus thickness across youth with PBD, but positively across HC.

**Conclusion:**

Neural correlates of LPP in youth included fronto-occipital areas that have been associated also with emotion processing and control. The opposite relationship between BPD and HC of LPP amplitude and right precentral gyrus thickness might explain the inefficacy of the emotional control system in PBD.

## 1 INTRODUCTION

Juvenile-onset bipolar disorder, also known as pediatric bipolar disorder (PBD), is a severe condition that has higher levels of chronicity, morbidity, suicidality, hospitalization, and social impairment than adult-onset bipolar disorder (BD) [1]. The pathophysiology of PBD is largely unknown, although emotion dysregulation has been proposed as a cardinal mechanism [2]. Emotion dysregulation can be investigated with tasks featuring emotional faces, words, or images [3-6]. Functional magnetic resonance imaging (MRI) techniques combined with tasks with emotional content have shown that youth with PBD had altered functioning in neural circuits associated with emotional recognition, appraisal, and control [7-10]. Accordingly, structural MRI reported smaller volumes and cortical thickening in areas belonging to this circuitry, such as the orbitofrontal, medial prefrontal, cingulate, dorsolateral, and ventrolateral prefrontal cortices, and altered volumes of deep gray matter structures such as the amygdala, [9-13]. Emotion regulation has been studied also with electroencephalography (EEG) using a specific event-related potential (ERP) named the late positive potential (LPP). The LPP has been proposed as a possible marker of emotional regulation [14, 15]. The LPP is a positive deflection beginning approximately 400 milliseconds after the presentation of an emotionally salient stimulus and is sustained through (and, briefly beyond) the end of the stimulus presentation [16]. The LPP reflects the activation of sustained attention towards emotionally salient stimuli and is sensitive to emotionally arousing faces, words, or pictures [17]. To date, the only available study on PBD was published by our group, revealing heightened LPP for positive stimuli compared to healthy controls (HC) [14]. In that study, we interpreted that finding as supporting evidence of poor emotion regulation in PBD, perhaps more specifically in relation to a propensity for mania. Neural sources of the LPP in PBD have not been investigated yet, and, also in healthy youth, are still unclear. This makes it difficult to place the findings obtained with MRI and the LPP within a single scientific model of PBD.

MRI techniques furnish information on possible neural circuits, but MRI is unable to elucidate the temporal dynamics of cerebral processing. EEG and ERPs have a high temporal resolution. MRI techniques complemented with ERP or EEG provide an opportunity to furnish a more comprehensive model of neural processing [18]. In healthy adults, sources of LPP have been located in areas involved in visual processing, attention, emotional regulation, and control, including the extrastriate occipital, inferotemporal, medial parietal, insular, anterior cingulate, orbitofrontal cortices [18-20]. Also, hyperactivation of deep gray matter structures involved in emotional processing, such as the amygdala, has been documented in adults [19, 20]. Only two studies investigated the LPP in healthy youth and those with anxiety. Wessing and colleagues [21] found that LPP amplitude elicited by angry faces positively correlated to heightened activity in the occipital cortex, parieto-temporal cortices, and ventrolateral prefrontal cortex. On the other hand, Bunford and colleagues [22] found that LPP elicited by fearful, angry, or happy faces was positively correlated to heightened activity of the inferior frontal gyrus, orbitofrontal cortex, left supplementary motor area, and right superior parietal lobule. To date, the effect of psychopathology on the LPP has been limited to anxiety symptoms, with inconsistent findings [21, 22]. No information is available if those findings translate to PBD. Furthermore, knowledge regarding neural sources of LPP is derived from EEG/functional MRI hybrid techniques, whereas there is an absence of studies investigating neuroanatomical counterparts of this ERP, such as cortical thickness and deep gray matter volumes.

We had three aims: i) to investigate the neuroanatomical correlates of the LPP in pediatric populations; ii) To further investigate LPP alterations in youth with PBD; iii) to relate LPP alterations to cortical thickness and deep gray matter volumes obtained by MRI. We chose cortical thickness, deep gray matter volumes, and LPP because of the relative independence of those outcomes from possible confounding variables, such as mood state [14, 23-25]. The LPP was measured with a passive viewing task containing emotional faces. Passive viewing tasks have been hypothesized to avoid biases related to task performance and to ease suitability for pediatric populations [26]. We hypothesized that compared to HC, i) youth with PBD would show reduced amygdala volume and reduced cortical thickness of orbitofrontal, cingulate, dorsolateral, and ventrolateral prefrontal cortices as evidence for a compromised network associated with emotion information processing; ii) youth with PBD would show a larger LPP amplitude for happy faces; iii) youth with PBD would show a lower or negative correlation between LPP amplitude and thickness of areas involved in emotional control; iv) since smaller amygdala volumes have been associated to greater amygdala activation to emotional stimuli [9], we expected that subjects with PBD show a lower or negative correlation between left and right amygdala volumes and LPP amplitude as compared to HC.

## 2 MATERIALS AND METHODS

This study was approved by the Baylor College of Medicine Institutional Review Board.

Patients with PBD were recruited from a child and adolescent outpatient psychiatric clinic. Patients’ parents/legal guardians signed informed consent and youth gave written assent before any study procedures were initiated.

### 2.1 Study Participants

The present study’s participants were 24 children and adolescents between 7-17 years old with PBD and 28 HC peers. The diagnosis of PBD followed DSM-5 guidelines [27], whereas the diagnosis of PBD-not otherwise specified (PBD-NOS) followed Course and Outcome of Bipolar Youth (COBY) research criteria [28]. Study participants underwent: (i) the Mini International Neuropsychiatric Interview-KID (MINI-KID) and the MINI-KID parent version [29] to determine psychiatric diagnoses; (ii) the Wechsler Abbreviated Scale of Intelligence - II (WASI-II) [30] to determine age- and sex-corrected general intelligence (composite IQ score); (iii) the Children Depression RatingScale-Revised (CDRS-R) [31] and the Young Mania Rating Scale (YMRS) [32] to determine the severity of depressive and manic symptoms at the time of testing; (iv) the emotional faces ERP paradigm to study dynamics and mental processes of emotional information processing; (v) three-Tesla MRI scan to study cortical thickness/deep gray matter volumes. Suitability for MRI scanning was required for all the youths to be included. Youth with PBD also required the absence of an eating disorder, ADHD, and anxiety disorders without comorbid PBD. HC also required: the absence of psychiatric illness and the absence of any psychiatric illness in first-degree relatives. Exclusion criteria for the whole sample include (i) comorbid substance use disorder; (ii) intellectual disability; (iii) comorbid autism spectrum disorder; (iv) severe neurological conditions.

### 2.2 Neuroimaging

The imaging protocol included a whole-brain T1-weighted scan acquired using a 3.0 T Siemens Trio scanner. Whole-brain T1-weighted images were obtained in the sagittal plane using the following sequence: TE/TR = 3.68/8.1 ms, matrix 256 × 256 × 180, voxel-size 1 × 1 × 1 mm3. Acquisition time lasted about 5 minutes.

#### 2.2.1 Cortical Thickness

Cortical thickness was computed for 34 bilateral Desikan-Killiany (DK) atlas regions [33] using FreeSurfer 6.0 standard, automated cortical reconstruction pipeline (http://surfer.nmr.mgh.harvard.edu/). The processing steps were as follows: (i) removal of non-brain tissue and transformation of the T1-weighted scans into the Talairach space; (ii) segmentation of deep white matter and gray matter anatomical volumes; (iii) motion correction and non-uniform intensity normalization [34]; (iv) grey/white matter tessellation, topology correction [35] and intensity gradient-based surface deformation to generate grey/white and grey/cerebrospinal fluid surface models [36-38]. The resulting surface models were then inflated and registered to a spherical surface atlas, allowing the parcellation of cortical regions of interest [35, 36, 38-40]. Finally, regional cortical thicknesses were computed by taking the mean of the white-pial distance at all vertices within each parcellated region [38].

#### 2.2.2 Deep Gray Matter Volumes

Deep gray matter volumes were determined using the automated procedure for volumetric measures of brain structures implemented in FreeSurfer 6.0. The automated procedure of deep gray matter volume segmentation has been described previously [41]. Briefly, this procedure automatically segments and labels each anatomic structure based on an atlas containing probabilistic information on the location of structures. This technique automatically assigns a neuroanatomical label to each voxel in an MRI volume based on probabilistic information estimated from a manually-labeled training set. The first stage is an affine registration with Talairach space followed by an initial volumetric labeling and correction from variation in intensity due to “intensity field bias”. Finally, a high dimensional non-linear volumetric alignment to the Talairach atlas is performed and the volume is labeled. The labeling uses an algorithm based on both a subject-independent probabilistic atlas and subject-specific measured values assigning a value at each point in the space using three types of probabilities: i) the probability of a given point belonging to each of the label classes; ii) the probability that a given point belongs to a label class given its neighboring points; and 3) the probability distribution function (for volume-based labeling is measured by the intensity at that voxel) of the measured value is estimated separately for each class at each point [41, 42]. This method provides advantages similar to manual region-of-interest (ROI) drawing [43, 44], without the risk of biases, offering an anatomically accurate rendering of regional volumes [45]. Intracranial volume (ICV) which includes biological material such as meninges and cerebrospinal fluid in addiction to brain tissue, was calculated to correct the regional brain volume analyses [46]. Specifically, we corrected the volume of each deep gray matter structure for ICV according to the proportion method [47]. For the present study, left and right gray matter volumes were estimated for the thalamus, amygdala, hippocampus, caudate nucleus, putamen, globus pallidus, and nucleus accumbens.

### 2.3 Neurophysiology

#### 2.3.1 ERP Paradigm

Participants were presented a passive emotional faces task. The paradigm and outcomes have been described previously [14]. The ERP paradigm was presented with E-Prime 2.0 and consisted of 180 consecutive trials. This paradigm used 10 fearful, 10 neutral, and 10 happy faces of children and adolescents that are included in the NIMH Child Emotional Faces Picture Set (https://devepi.duhs.duke.edu/nimh-chefs-picture-set/), and 10 fearful, 10 neutral, and 10 happy faces of adults that are included in the NimStim Set of Facial Expressions (www.macbrain.org/resources.htm). The faces were balanced with respect to sex and race/ethnicity (Caucasian, African-American, and Hispanic). The final stimulus set consisted of 60 greyscale pictures of male or female youth or adults with fearful, happy, or neutral facial expressions. Each of the 180 trials started with a 500-ms fixation cross presented in the middle of a computer screen. The fixation cross was followed immediately by a 1000-ms duration picture of a face. Faces were 17 cm high and 15 cm wide on a 27-inch diagonal flat-screen computer monitor. Participants sat in a comfortable chair about 34 cm from the screen and were instructed to passively look at each picture and to keep their eyes focused on the location of the fixation cross. Each face appeared 3 times during the 180 trials in this paradigm. The presentation of face pictures was randomized with the exception that the same picture was never presented consecutively.

#### 2.3.2 LPP Recording and Processing

Raw EEG signals were recorded with BrainVision Recorder (Brain Products GmbH) from 32 active electrodes following the 10-20 international system of EEG electrode placement (ActiCAP; Easycap GmbH). AFz served as ground; FCz served as reference. Impedances were below 20 kΩ. Signals were sampled at 250 Hz, filtered between 0.1-1000 Hz, and amplified with a BrainAmp Standard amplifier (Brain Products GmbH).

Signals were analyzed offline with EEGLAB [47] and ERPLAB [48]. Raw signals were re-referenced to TP9/10, epoched between 200 ms pre-stimulus to 1500 ms post-stimulus relative to stimulus presentation, 0.1-30 Hz band-pass filtered, and corrected for baseline. Trials with incidental, non-repetitive artifacts (*e.g*., clipping; movement) were manually rejected prior to independent component analysis (ICA; runica option in EEGLAB) [49]. ICA components representing artifacts (eye blinks, eye movements, EKG, channel pop, drift) were removed from the signal. Next, the signals were baseline corrected and averaged per stimulus type, obtaining 6 ERPs per subject (3 emotions, *i.e*., fearful, neutral, happy, for 2 sets, *i.e*. youth, adult). The LPP was measured as the average amplitude between 400-1000 ms post-stimulus, averaged across O1, O2, and Oz which is the appropriate location to obtain the LPP for the age range of the participants in this study [50].

### 2.4 Statistical Analyses

All the analyses were performed using SPSS software version 21.0 (IBM, Somers, NY).

#### 2.4.1 Demographics

Between-group differences for demographic and clinical variables were investigated with multiple *t*-tests and chi-square tests.

#### 2.4.2 Brain Structure

Because of the different nature of brain’s morphometric indices (thickness and volumes), separate statistics were performed for cortical thickness and deep gray matter volumes while calculating between-group differences.

##### 2.4.2.1 Cortical Thickness

Because of the high cortical thickness parcellation, principal component analysis (PCA) was performed across all subjects to reduce variable dimensionality and to identify regional cortical thickness clustering. PCA reduces large sets of variables to a few minimally-associated vectors of weightings that best explain the variance across variables while losing as little information as possible. Each extracted vector, referred to as a “component”, accounts for a portion of the total variance in the data: the first component accounts for the largest amount of variance, with each successive component accounting for a smaller amount of the total variance. PCA was conducted across 64 regional cortical thicknesses across all subjects. The optimal number of components was determined *via* scree plot. Loadings exceeding .400 or -.400 were considered for data interpretation. If variables exceeded this threshold for more than one component, only the component with the highest loading was considered. However, if small differences between two significant loadings were present, the variable was not considered for data interpretation. After component identification, each subject’s component score was generated for each component retained. These components were further regressed using groups (PBD, HC) as dependent variables. Significance was set to <.05.

#### 2.4.3 Deep Gray Matter Volumes

To reduce type I errors, two separate multivariate analyses of variance (MANOVAs) were performed. In each MANOVA, groups were independent variables, whereas left- and right-deep gray matter volumes were dependent variables. Outcomes were corrected for multiple comparisons (Bonferroni) when needed. In the case of a significant MANOVA, multiple one-way t-tests were performed to investigate differences in deep gray matter volumes. In each t-test, groups (PBD, HC) were independent variables, whereas left- and right-deep gray matter volumes were dependent variables. Bonferroni correction was also applied for multiple comparisons.

#### 2.4.4 LPP

Between-group differences were investigated using repeated measures ANOVAs using group (2 levels: PBD, HC) as a between-subjects variable, and set (2 levels: youth, adult faces) and emotion (3 levels: fearful, neutral, happy expressions) as within-subjects variables. Significant main effects and interactions were examined with additional repeated measures. Within-subject effects were corrected for sphericity violations using the Greenhouse-Geisser algorithm. Outcomes were considered significant if *p* <.05. Outcomes were corrected for multiple comparisons (Bonferroni) when needed.

#### 2.4.5 Relationship between Cortical Thickness/Deep Gray Matter Volumes and LPP Amplitude

Multiple linear regressions were performed to investigate relationships between cortical thickness/deep gray matter volumes and LPP amplitude. In each regression, LPP amplitude was used as the outcome variable. Predictor variables included group (PBD, HC), components retained in the PCA, each deep gray matter volume, and cortical thickness/deep gray matter volumes by group interactions. Outcomes were considered significant if *p* <.05. Multiple comparisons correction was applied when needed.

## 3 RESULTS

### 3.1 Demographics

24 youths with PBD and 28 HC were included in the analyses. Demographic and clinical variables are displayed in Table **1**. Subjects with PBD were moderately hypomanic or depressed. Since measurements of either LPP, cortical thickness and subcortical volumes have been shown to not be influenced by mood state [14, 23-25], these variables were not included in the following analyses.

### 3.2 Cortical Thickness

The scree plot suggested three principal components (PC). The fourth PC accounted for less than 5% of the variance, therefore, this and the following PCs were not retained. A graphical representation of PCs is presented in Fig. (**1**). The first PC (PC1) identified a broad area mainly encompassing temporal regions, including left and right banks of the superior temporal sulcus, left and right fusiform, left and right inferior, middle and superior temporal, right transverse temporal gyri, and the right insula. PC1 also included parts of the parietal and frontal lobes such as the right posterior cingulate and right caudal middle frontal gyri. The second PC (PC2) corresponded to areas belonging mainly to occipital and frontal lobes, including left and right cuneus, left and right lateral occipital and peri calcarine cortices, left and right caudal anterior cingulate, left and right lingual, left and right postcentral, left superior frontal, left and right rostral middle frontal gyri and the left and right frontal poles. The third PC (PC3) revealed a narrower set of areas corresponding to the left and right isthmus of the cingulate gyrus, the left and right precentral cortex, the left caudal middle frontal gyri, and the right medial orbitofrontal cortex. Regression analyses revealed group differences in the PC3 (R^2^ = .07, *p* = .045), which showed greater cortical thickness for HC than PBD. No differences were found for PC1 (R^2^ = -.01, *p* = .413) and PC2 (R^2^ = .01, *p* = .306).

### 3.3 Deep Gray Matter Volumes

The MANOVA reported no significant group effect for either the left (Wilk’s Lambda = .90; F = .50; DF = 7; *p* = .827) or right (Wilk’s Lambda = .89; F = .55; DF = 7; *p* = .792) deep gray matter volumes. Therefore, t-tests were not performed.

### 3.4 LPP

There was a main effect of group (F = 4.25; *p* = .045) and a main effect of set (F = 1.18; *p* = .046), whereas effects of emotion, group by emotion, and set by emotion interactions were not significant (F = .61, *p* = .940; F = .46, p = .635; F = 1.56, *p* = .214, respectively). As regards the group's main effect, subjects with PBD showed greater LPP amplitude than HC. Despite the non-significance of the emotion by group interaction, in order to explore the possible driving effect of emotion in between-group differences, post-hoc analyses were performed for such interaction. Post-hoc tests revealed that the group effect was mainly driven by the LPP elicited by happy faces. As regards the effect of set, adult faces elicited greater LPP than Childs’ faces, irrespective of emotions and without a group effect. Group effects and post-hoc comparisons are present in Table **2**.

### 3.5 Relationship between Cortical Thickness/Deep Gray Matter Volumes and LPP Amplitude

Results of linear regressions are present in Table **3** and Fig. (**2**). Regressions revealed a significant effect of the PC2 and the right globus pallidus volume on the overall LPP amplitude (average across 400 to 1000 ms post-stimulus). Additionally, a group by right precentral gyrus thickness interaction effect was found. Post-hoc analysis revealed that the thickness of areas belonging to the PC2 positively correlated to the LPP amplitude whereas the right globus pallidus volume negatively correlated with the LPP amplitude. As regards the interaction effect, HC showed a positive correlation between the thickness of the right precentral gyrus and LPP amplitude, whereas in PBD this correlation was negative.

Since between-group differences in the LPP were mainly driven by happy faces, exploratory regressions were performed considering only the LPP elicited happy faces across all subjects. A significant main effect was observed for PC2 and right globus pallidus volume. Additionally, a main effect was observed for the left caudate and left globus pallidus. There was a positive correlation between PC2 and LPP amplitude elicited by happy faces, whereas the correlation between LPP and left and right globus pallidus and right caudate were negative. Interaction effect and differential correlations in youth with PBD and HC were identical to those found for the overall LPP.

## 4 DISCUSSION

Results might be summarized as follows: i) youth with PBD showed a thinner left and right isthmus of the cingulate gyrus, the left and right precentral gyri, the right medial orbitofrontal and left caudal middle frontal cortices than HC; ii) youth with PBD showed greater LPP amplitude than HC which was mainly driven by LPP elicited by happy faces; iii) greater LPP amplitude was associated with greater thickness in areas belonging to a broad fronto-occipital network and smaller right globus pallidus volume; iv) neural correlates of LPP elicited by happy faces overlap those of the LPP elicited by all emotional faces. Additionally, a positive relationship between LPP for happy faces and right globus pallidus and right caudate emerged; v) differences in between-group LPP/cortical thickness correlations slopes involve the right precentral gyrus: while HC showed a positive correlation between the right precentral gyrus thickness and LPP elicited by all the emotional and the happy faces, youth with PBD showed a negative correlation.

The present findings corroborate the available evidence of MRI structural alterations in frontal areas involved in emotional control, such as the orbitofrontal cortex [11, 12] and the dorsolateral prefrontal cortex, which embeds the caudal middle frontal gyrus, in youth with PBD [51]. The present findings are also in line with those documenting PBD-related structural alterations in the precentral gyrus, whose function extends beyond movement and include regulatory functions over emotions [52], and with those reporting adult-BD-related functional alterations of the posterior cingulate cortex, an area involved in mood symptoms such as anhedonia and affective flattening [53]. Additionally, results corroborate previous findings documenting greater LPP amplitude in PBD than in healthy peers [14]. Such difference is mainly driven by happy faces, and suggests heightened sustained attention towards positive emotion, *i.e*. a positive bias. Positive bias has been already demonstrated through behavioral tasks using go/no go paradigms [54, 55] and has been proposed as a marker of predisposition to mania [56] and an endophenotype of bipolar disorder [57]. The majority of the present study’s participants have a BD type I or Bipolar NOS I diagnosis. BD type I is the BD subtype in which mania represents the most severe manifestation, and which embeds a tendency to develop manic episodes [58, 59]. Though behavioral manifestations are milder, the psychopathology of youth with BD-NOS resembles the one of BD, type I [28]. Therefore, heightened LPP in this sample might represent the neural mechanisms underlying this predisposition.

Greater LPP is positively correlated with thickness in several areas mainly belonging to the prefrontal and occipital lobes. Also, positive correlations with some areas belonging to the parietal lobe are represented. These findings are substantially in line with studies in children and adults and confirm that neural sources of the LPP rely on areas belonging to occipital extrastriate cortices that are involved in bottom-up processing of visual stimuli, and prefrontal areas involved in either automatic and voluntary top-down control of emotions [18-22]. The involvement of the post-central gyrus might explain the parietal components of the LPP involved in attention. Even though the post-central gyrus is mainly involved in somatosensory processing, its activation is involved in the allocation of attentional resources during visual across multiple stimuli [60]. More specifically, the precentral gyrus is embedded in a broad prefrontal-parietal network involved in a top-down, voluntary reorientation of attentional resources and in the detection of task-relevant salient stimuli outside the direct focus of attention [61]. This network includes prefrontal areas, such as the middle frontal gyrus and superior frontal gyrus, whose thickness has been shown to correlate with the LPP [60]. Therefore, the correlation we found between the thickness of these cortical areas and the LPP might be an indirect measure of activation of this prefrontal parietal network.

In contrast with our a priori hypothesis, we did not find any correlations between LPP amplitude and amygdala volumes. Instead, we found a negative correlation between LPP amplitude for happy faces and bilateral globus pallidus and left caudate volumes. This finding is in contrast with studies of Sabatinelli and colleagues and Liu and colleagues [19, 20], who found a coupling between emotionally-related LPP and amygdala activity. Differently from these studies, the present work assessed amygdala volume, whereas the others focused on amygdala function. Amygdala functional activity might not be captured by volumetric assessment, especially in youth with PBD [10] and therefore, the absence of volumetric alterations in PBD compared to HC might not signify an absence of hypo- or hyperactivity. On the other hand, findings regarding the negative relationship between bilateral globus pallidus and left caudate and LPP amplitude has been not been documented before. The caudate and the globus pallidus participate in emotional face processing, and activation of these two structures have been related to happy faces [62, 63]. These two structures are part of motivational systems [62, 64] and highly interconnected with prefrontal areas [62, 64]. The globus pallidus relates to the linkage between hedonic appreciation (a person’s ability to judge the pleasantness of a stimulus) and motivated action, with lesions resulting in a reduced ability to generate positive emotion and/or convert positive emotional states into action [65]. The caudate is involved in the subjective, affective meaning of motor actions, which in turn promotes the interpretation/appraisal process during the search for the most likely cause of the bodily changes [66], especially for reward-related information and goal-directed behavior [67-69]. The existing knowledge that links small deep gray matter structure volume and hyperfunction [9, 70] led to a hypothesized greater activation of these areas in the context of affective processing. Such hyperactivation might result in either greater hedonic drive or misinterpretation of affective cues to detriment of negative valence akin to positive bias that we discussed in a previous paragraph. This activation might turn into heightened LPP for emotional faces and specifically for happy faces.

Even though correlations among LPP and cortical thickness/deep gray matter volumes in PBD and HC go in the same direction, we found significant between-group differences in the relationship between LPP amplitude and the precentral gyrus thickness: HC showed a positive correlation, PBD showed a negative correlation. Exploratory analyses revealed that this correlation was also present for happy faces. The precentral gyrus is involved in the modulation of motivation and reward [71, 72] and goal-directed action control [73, 74]. The right precentral gyrus has been associated with the modulation of sustained attention [75], self-related awareness [76], and greater use of cognitive reappraisal [77]. Therefore, it can be inferred that reduced thickness in this area, which may reflect poor precentral gyrus activation, might result in poor cognitive control and poor modulation of sustained attention for emotionally salient stimuli in PBD. This might turn into heightened recruitment of attentional resources for emotive cues and poor emotional control. This imbalance might be captured by the LPP and might explain the heightened LPP amplitude in youth with PBD as compared to HC.

## 5 LIMITATIONS

The main limitation of the present study resides in the fact that MRI and EEG recordings are not performed at the same time, even though measurements were performed within 24 hours. Furthermore, the present study’s measurements were limited to brain structure and did not account for brain function. Since measurement of LPP represents a task-related, functional neurophysiological correlate of brain functioning, combining functional MRI (MRI), rather than structural MRI (MRI), with ERP techniques, might confer greater reliability to any results linking neuroimaging and neurophysiology. Therefore, the present study’s design should be considered non-optimal. As a consequence, hypotheses made in the discussion on mechanisms underlying the LPP should be considered speculative, as structural brain alterations represent an indirect measure of hyper-function/ dysfunction of a certain brain area. Further studies combining fMRI and EEG techniques are warranted to clarify the relationship between the LPP and its neural correlates in PBD. Further uncertainty around the generalizability of the results is brought about by the present study’s small sample size. Larger sample sizes are needed to clarify the neural sources of the LPP, in particular, if there are biotypes of PBD that are characterized by different neural circuits driving the LPP. Additionally, the present study did not account for the effect of additional, possible variables that might influence brain structure and function, such as age [78], gender [79], psychotropic medications [80-83], cyclicity [58], and polarity [84, 85].

## CONCLUSION

The present findings add new knowledge on regional sources of LPP, integrating neuroanatomical findings to already existing literature coming from functional MRI. Areas associated with LPP, *i.e*., extrastriate cortices, prefrontal and parietal cortices, sustains the putative role of the LPP as a marker of emotion regulation and attentive processes towards visually, emotionally-arousing stimuli. Between-group differences in LPP suggest biases towards positive emotions in PBD. Regression analyses suggest that such positive bias might be related to the altered function of the post-central gyrus. Specifically, reduced thickness of the precentral gyrus, which may reflect poor activation, might lead to reduced control of attentional resources toward positive stimuli. This might turn in heightened arousal and poor emotional control into positively-valenced emotions. The neurophysiological counterpart of emotional dyscontrol is the heightened LPP in PBD. Understanding the anatomical counterpart of the LPP would help the development of reliable markers of disease onset and progression. More specifically, since altered bias towards positive stimuli represents a possible marker for developing manic states, the precentral gyrus might surge as a possible marker of such fragility, thus helping the early recognition of subjects with high risk to develop PBD or relapses through MRI techniques. Additionally, targeting the precentral gyrus with physical treatments, such as transcranial magnetic stimulation, might confer protection against mood destabilization. Nevertheless, research on neuromarkers and targeted brain treatments are still in its infancy. Additional studies are needed to reveal the neurobiology of mood dysregulation in PBD.

## Figures and Tables

**Fig. (1):**
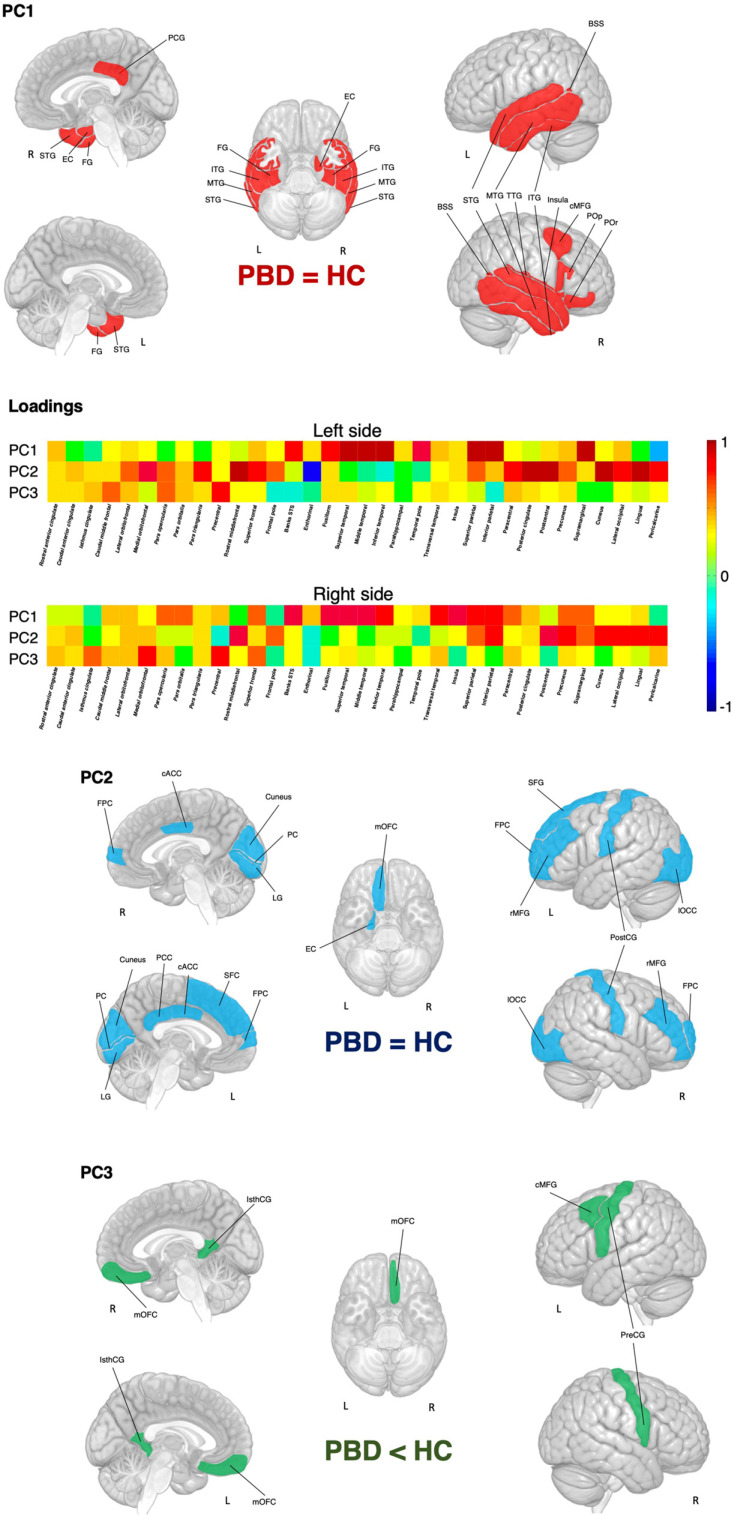
Cortical thickness loadings and PCs. **Abbreviations:** PC: principal component. PBD: pediatric bipolar disorder; HC: healthy controls. BSS banks of the superior temporal sulcus, cACC: caudal anterior cingulate, cMFG: caudal middle frontal gyrus, ITG: inferior temporal gyrus, IsthCG, isthmus of the cingulate gyrus, FG: fusiform gyrus, FPC: frontal pole cortex, LG: lingual gyrus, lOCC: lateral occipital cortex, mOFC: medial orbitofrontal cortex, PC: pericalcarine cortex, PCG: posterior cingulate gyrus, PreCG: precentral cingulate gyrus; PostCG, post central gyrus, rMFG: rostral middle frontal gyrus, SFG: superior frontal gyrus, STG: superior temporal gyrus, TTG: transverse temporal gyrus.

**Fig. (2):**
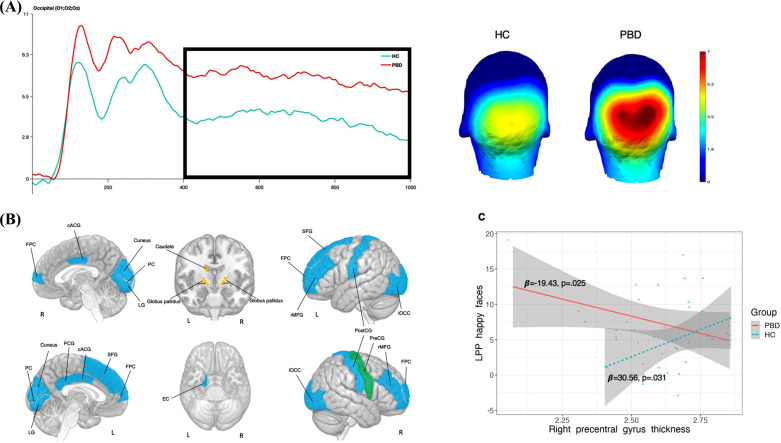
Neural correlates of LPP. (**A**) LPP amplitude elicited by happy faces in youth with PBD and HC; (**B**) brain areas related to LPP for happy faces in youth with LPP and HC; V) relationship between right precentral gyrus thickness and LPP elicited by happy faces in youth with PBD and HC. Brain areas belonging to the PC2 are colored in blue; areas belonging to the PC3 are colored in green; deep gray matter volumes are colored in yellow. **Abbreviations:** LPP: late positive potential. PBD: pediatric bipolar disorder; HC: healthy controls. cACC: caudal anterior cingulate, FPC: frontal pole cortex, LG: lingual gyrus, lOCC: lateral occipital cortex, PC: pericalcarine cortex, PCG: posterior cingulate gyrus, PreCG: precentral gyrus; PostCG, postcentral gyrus, rMFG: rostral middle frontal gyrus, SFG: superior frontal gyrus.

**Table 1:** Sociodemographic and clinical characteristics of youth with PBD and HC.

-			**PBD (*N* = 24)**	**HC (*N* = 28)**	***F* or χ^2^**	***p-value***
**Demographics**						
Age (y), mean ± SD			11.96 ± 3.10	11.86 ± 3.11	.01	.907
Female, n (%)			13(54.16)	13(46.43)	.31	.578
Race, n (%)			-	-	-	-
*Asian*			0(0.00)	2(7.10)		
*African-American*			2(8.30)	6(21.40)	3.81	.149
*Caucasian*			22(91.70)	20(71.40)		
**Ethnicity**						
*Hispanic*			2(8.30)	3(7.10)	.026	.872
I.Q., mean ± SD			98.96 ± 17.58	103.30 ± 12.99	1.02	.318
**Clinical**						
*Year ill (y), mean ± SD*			3.64 ± 2.36	-	-	-
*CDRS, mean ± SD*			31.22 ± 12.27	17.84 ± 2.08	30.32	**<.001**
*YMRS, mean ± SD*			2.54 ± 3.32	14.22 ± 10.58	27.72	**<.001**
**PBD Type**						
*Type 1*			17(70.80)	-	-	-
*Type II*			0(0.00)	-	-	-
*Not otherwise specified*			7(29.20)	-	-	-
Mood State*						
*(hypo)manic*			7(41.20)	-	-	-
*Depressed*			4(23.50)	-	-	-
*Euthymic*			6(35.30)	-	-	-
**Comorbidity, n (%)**						
*None*			8(33.30)	-	-	-
*ADHD*			9(37.50)	-	-	-
*ODD*			2(8.30)	-	-	-
*Panic Disorder*			1(4.20)	-	-	-
*GAD*			4(16.70)	-	-	-
**Current Pharmacotherapy, n (%)**						
*AD*			13(66.70)	-	-	-
*AP*			11(45.80)	-	-	-
*MS*			11(45.80)	-	-	-
*BDZ*			0(0.00)	-	-	-
*MARI*			13(54.20)	-	-	-

**Note**: Significant *p*-values (*p* < 0.05) are indicated in bold, trend-level *p*-values (*p* < .07) are indicated in italics. **Abbreviations**: PBD: youth with bipolar disorder; HC, healthy controls; ADHD, attention deficit hyperactivity disorder; ODD, oppositional-defiant disorder. AD, antidepressant; AP, antipsychotic; MS, mood stabilizer; BDZ, benzodiazepines; MARI, mixed monoamine reuptake inhibitor.

*Mood state is calculated for youth with PBD type 1.

**Table 2:** LPP amplitude in subjects with PBD and HC.

**Group**		**pBD (*N* = 24)**	**HC (*N* = 28)**	***F***	***p-value***
LPP (PO1, PO2; POZ)					
*Fearful, μV, mean* ± SD		7.29 ± 4.81	5.14 ± 5.49	2.17	.147
*Neutral, μV, mean* ± SD		7.39 ± 5.10	5.03 ± 4.30	3.29	.076
*Happy, μV, mean* ± SD		7.58 ± 4.19	4.56 ± 4.62	6.00	**.018**
**Set**		**Child Faces**	**Adult Faces**	-	-
*Overall, μV, mean* ± SD		5.72 ± 4.50	6.42 ± 4.87	1.18	**.046**

**Note**: Significant *p*-values (*p* < 0.05) are indicated in bold, trend-level *p*-values (*p* < .07) are indicated in italics. **Abbreviations**: PBD: youth with bipolar disorder; HC, healthy controls; SD: standard deviation.

**Table 3:** Association between cortical thickness/subcortical volumes and LPP amplitude in the whole sample (PBD + HC).

**Areas**	**Thickness Main Effect (All Faces)**		**Thickness Main Effect (Happy Faces)**		**Thickness by Group ** **Interaction (All Faces)**		**Thickness by Group ** **Interaction (Happy Faces)**	
	**F**	**p**	**F**	**p**	**F**	**p**	**F**	**p**
PC1	2.75	.106	.41	.523	.611	.439	1.64	.687
PC2	**4.91**	**.033**	**5.55**	**.024**	.01	.934	.06	.805
L-isthmus cingulate	1.08	.304	.18	.673	.02	.883	.39	.539
R-isthmus cingulate	.02	.883	1.41	.243	.74	.396	.04	.848
R-precentral	.70	.407	.26	.614	**5.97**	**.019**	**5.21**	**.028**
L-precentral	.32	.373	.16	.696	1.81	.187	.93	.341
R-medial orbitofrontal	1.63	.209	1.31	.260	.11	.742	.07	.798
L-caudal midlle frontal	.64	.429	1.63	.210	.71	.406	1.22	.276
L-amygdala	1.02	.318	1.16	.289	<.01	.973	.01	.911
R-amygdala	1.40	.245	1.81	.187	.33	.570	.13	.725
L-caudate	*3.96*	*.054*	**4.69**	**.037**	.31	.584	.38	.542
R-caudate	1.87	.180	2.77	.105	.05	.826	.2.77	.105
L-globus pallidus	2.21	.146	**4.44**	**.042**	.14	.706	1.29	.264
R-globus pallidus	**4.32**	**.045**	**5.80**	**.021**	.15	.698	1.08	.307
L-hippocampus	6.65	.420	1.06	.331	.32	.576	.64	.428
R-hippocampus	.98	.329	1.12	.297	.28	.599	.79	.380
L-putamen	.74	.394	.02	.887	.11	.744	.02	.887
R-putamen	.13	.724	.69	.413	.20	.657	<.01	.969
L-nucleus accumbens	2.36	.630	.15	.698	.66	.421	1.83	.185
R-nucleus accumbens	.99	.326	.58	.452	1.10	.302	2.15	.152
L-thalamus	3.37	.075	*3.75*	*.061*	.04	.851	.13	.718
R-thalamus	2.57	.118	2.70	.109	.02	.877	.08	.774

**Legend**: Significant *p*-values values (*p* < 0.05) are indicated in bold, trend-level *p*-values (*p* < .07) are indicated in italics. **Abbreviations**: LPP: late positive potential; PC: principal component. PBD: youth with bipolar disorder; HC, healthy controls.

## Data Availability

Not applicable.

## LIST OF ABBREVIATIONS

BD: Bipolar Disorder
EEG: Electroencephalography
ERP: Event-related Potential
HC: Healthy Controls
ICV: Intracranial Volume
LPP: Late Positive Potential
MRI: Magnetic Resonance Imaging
PBD: Pediatric Bipolar Disorder
PCA: Principal Component Analysis
ROI: Region-of-Interest
